# Defining the Response of Caenorhabditis elegans to Multiple Simultaneous Stressors

**DOI:** 10.1093/geroni/igaa057.405

**Published:** 2020-12-16

**Authors:** George Sutphin, Hope Dang, Emily Turner, Destiny DeNicola, Sage Hamming, Amanda Furtmann, Delaney Kelser

**Affiliations:** University of Arizona, Tucson, Arizona, United States

## Abstract

Cells are constantly subjected to a variety of intrinsic and extrinsic stresses—oxidative, protein misfolding, osmotic—and respond by activating a range of molecular pathways to mitigate and repair damage—oxidative stress response, unfolded protein response, osmotic stress response. While individual stress response pathways have been described in detail, and some interventions improve resistance to multiple forms of stress (e.g. dietary restriction, insulin signaling inhibition), surprisingly little is known about how these responses differ when cells are challenged with multiple types of stress simultaneously. The molecular architecture underlying multi-stress response has broad implications for aging and age-associated disease. One characteristic of aging is a progressive increase in multiple categories of cellular stress accompanied by a decline in cellular stress response capability. Human diseases rarely involve a single form of stress—Alzheimer’s disease is characterized by neuroinflammation, oxidative stress, and misfolded proteins, while cancer exhibits oxidative stress, DNA damage, and localized hypoxia. Determining how cells respond differently to one form of stress in the presence of another is critical to building an accurate model of the aging cellular environment. We are using Caenorhabditis elegans to systematically evaluate the molecular network that cells employ when challenged by multiple simultaneous stressors and how different stress combinations impact organismal survival and health. Here we present our initial characterization of C. elegans response to multiple categories of cellular stress (oxidative, osmotic, ER, Golgi, heavy metal), which stressors elicit non-additive interactions when combined, and how these combinations impact survival, health, and established stress response pathways.

